# Arteriovenous malformation of the spermatic cord as the cause of acute scrotal pain: a case report

**DOI:** 10.1186/1752-1947-1-110

**Published:** 2007-10-16

**Authors:** Petros Sountoulides, Athanasios Bantis, Irene Asouhidou, Hellen Aggelonidou

**Affiliations:** 1 Department of Urology, University Hospital of Alexandroupolis, Dragana 68100, Alexandroupolis, Greece; 2 Department of Anaesthesiology, University Hospital of Alexandroupolis, Dragana 68100, Alexandroupolis, Greece; 3 Department of Pathology, University Hospital of Alexandroupolis, Dragana 68100, Alexandroupolis, Greece; 4 15-17 Agiou Evgeniou street, 55133, Thessaloniki, Greece

## Abstract

Arteriovenous malformations of the lower urinary tract are uncommon lesions, usually presenting as scrotal masses. A case of recurrent acute scrotal pain mimicking testicular torsion that was attributed to the presence of an arteriovenous malformation of the spermatic cord is described. To our knowledge this is the first reported case of an arteriovenous malformation of the spermatic cord presenting with acute scrotal pain.

## Background

### Introduction

Arteriovenous malformations (AVMs) occur mainly in the central nervous system, although they have been described in other organs as well. AVMs rarely involve the testis or the scrotal components, presenting mainly in the form of para-testicular or intra-testicular masses. This case reveals an uncommon clinical presentation of a non-palpable AVM of the spermatic cord. To our knowledge we present the first reported case of an arteriovenous malformation of the spermatic cord being the cause of recurrent acute scrotal pain.

### Case presentation

An otherwise healthy 22-year-old man presented with acute pain in his right hemiscrotum. He recalled having similar episodes of self-limited scrotal pain since his adolescence. The pain had been attributed by attending physicians to episodes of intermittent testicular torsion. He denied any history of trauma, urinary tract infection, sexually transmitted diseases, or voiding symptoms.

Clinically both testes were intrascrotal, there was no swelling or signs of inflammation and the cremasteric reflex was intact. The right testis was, however, very tender in palpation. Both epidydimis were also normal and non-tender in palpation. Routine laboratory test and urinalysis were normal, and urine culture was sterile.

**Figure 1:**
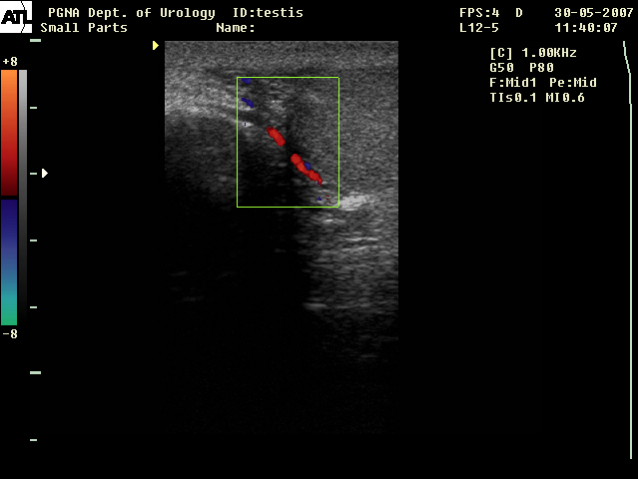
Color Doppler Ultrasound of the right hemiscrotum showing normal testicular parenchyma and blood flow.

Grey scale scrotal ultrasonography and Color Doppler Ultrasound (CDU) were performed. Sonography revealed no evidence of testicular tumor, varicocele or other pathology of the testis or epidydimis. (Figure 1)

The patient underwent a diagnostic right scrotal exploration due to his persistent scrotal pain. Scrotal exploration revealed a macroscopically normal testicle, epididymis and spermatic cord. There was no evidence suggestive of intermittent testicular torsion, e.x "bell clapper" deformity of the testis, or torsion of testicular appendages.

**Figure 2:**
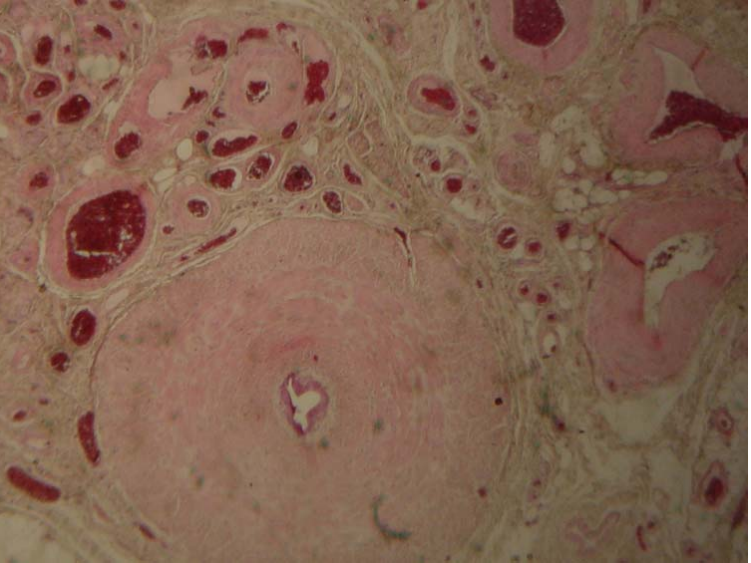
Arteriovenous malformation characterized by proliferation of arteries. Venous component exhibits several dilated vessels. Van Gieson's stain, reduced from ×100.

Four months later the patient presented with the same intractable right scrotal pain. Clinical examination and ultrasound investigation were insignificant. Orchiectomy was suggested this time and was performed through an inguinal incision after obtaining patient's consent. The surgical specimen consisted of a 16 ml testicle with its spermatic cord structures. The testis and spermatic cord were macroscopically unremarkable. Detailed pathology examination of the testicle and vas deferens revealed insignificant pathological changes, except for a small, smooth vascular mass (0.5 cm maximum diameter) in the lower part of the spermatic cord with the characteristics of an arteriovenous malformation (Figure 2). The patient was uneventfully discharged the following day. The patient has not experienced another episode of scrotal pain during the last 2 years.

### Discussion

Arteriovenous malformations (AVMs) represent defects of the circulatory system that are generally believed to arise during embryonic or fetal development or soon after birth. The characteristic of AVMs is that arteries and veins are tangled and not connected by capillaries. The lack of capillaries allows blood traveling through these abnormal vessels to flow rapidly and under high pressure, thus preventing arterial blood from reaching the tissues leading to various degrees of ischemia and resulting pain. Histologically, the irregular vascular spaces are lined by nonproliferating and quiescent endothelial cells and are separated by fibrous stroma [1]. The abnormal vascular tissue within these malformations is predominantly of type 1 (arterial, venous or lymphatic) or a combination of vascular types [2].

AVMs involving the lower urinary tract are uncommon as opposed to AVMs located in the Central Nervous System (CNS). Even more rare is the case of an AVM of the urinary tract presenting with pain. There is a report of ejaculatory pain caused by an AVM located between the prostate and seminal vesicles [3], while there is only one report of a renal AVM presenting with chronic flank pain without hematuria [4].

Arteriovenous malformations of the spermatic cord and testis are benign lesions consisting of complex tangles of enlarged dilated arteries and veins without intervening capillaries. In the cases published so far AVMs of the scrotal compartments present as either painless paratesticular masses [2,5-7] or as incidental findings during evaluation for infertility [8] or as combination of both infertility and scrotal swelling [9].

Scrotal AVMs appearing as masses can be detected by pelvic arteriography and managed by subsequent superselective embolization, however success is not always guaranted [9], necessitating open surgical excision of the lesion.

However in our case the small size of the lesion would make it impossible to detected by means of arteriography and to embolize successfully. The decision for orchiectomy was justified by the patient's intractable pain, suggesting some degree of testicular torsion considering his age, and the absence of findings on imaging studies.

### Conclusion

The case presented highlights a rare and unique cause of recurrent acute scrotal pain, attributed to the presence of an arteriovenous malformation of the spermatic cord. Therefore there is reason to believe that AVMs of the scrotum should be considered in the aetiology of otherwise inexplicable, recurrent scrotal pain. In that case consideration should be given to a trial of superselective angio-embolization of the lesion before one resorts to orchiectomy.

## Competing interests

The author(s) declare that they have no competing interests.

## Authors' contributions

PS was the treating urologist of the patient and performed together with AB both surgical procedures. PS has also drafted the manuscript. IR was the anaesthesiologist in charge in both operations performed. HA was the pathologist performing the stains and making the final pathology report for the case.

All authors have have read and approved the final manuscript.

## References

[B1] Bumpers PM Hulbert WC Jimenez JF Arteriovenous malformation of the spermatic cord J Urol 1989 141 1 103 4 2908929 10.1016/s0022-5347(17)40605-7

[B2] Guz BV Ziegelbaum M Pontes JE Arteriovenous malformation of spermatic cord Urology 1989 33 5 427 8 10.1016/0090-4295(89)90042-3 2775370

[B3] Angunawela RM Shepherd DF Hayward MJ De Silva AH Ejaculatory pain associated with a pelvic arteriovenous malformation Sex Transm Infect 2001 77 5 385 10.1136/sti.77.5.385 11588288 PMC1744359

[B4] Wong C Leveillee RJ Yrizarry JM Kirby K Arteriovenous malformation mimicking a renal cell carcinoma J Endourol 2002 16 9 685 86 10.1089/089277902761403050 12490024

[B5] McCracken JM MacNeily AE Mueller D Magee F Ultrasound features of a paratesticular arteriovenous malformation: a case report of an 11-year-old boy Pediatr Radiol 2005 35 5 532 4 10.1007/s00247-004-1358-6 15549283

[B6] Kang TW Choi YD Jeong YY Kwon DD Park K Ryu SB Park YI Intrascrotal extratesticular arteriovenous malformation Urology 2004 64 3 590 15351608 10.1016/j.urology.2004.04.040

[B7] Oktay B Ozyurt M Erol O Simsek U Arteriovenous malformation of the spermatic cord Br J Urol 1991 67 2 216 2004242 10.1111/j.1464-410x.1991.tb15116.x

[B8] Skiadas V Antoniou A Primetis H Moulopoulos L Vlahos L Intratesticular arteriovenous malformation. Clinical course, ultrasound and MRI findings of an extremely rare lesion on a 7 year follow-up basis Int Urol Nephrol 2006 38 1 119 22 10.1007/s11255-005-0916-7 16502065

[B9] Monoski MA Gonzales RR Thomas AJ Goldstein M Arteriovenous malformation of scrotum causing virtual azoospermia Urology 2006 68 1 203 e5–6 16808963 10.1016/j.urology.2006.01.019

